# Challenges and Approaches to Genotyping Repetitive DNA

**DOI:** 10.1534/g3.119.400771

**Published:** 2019-11-22

**Authors:** Elizabeth A. Morton, Ashley N. Hall, Elizabeth Kwan, Calvin Mok, Konstantin Queitsch, Vivek Nandakumar, John Stamatoyannopoulos, Bonita J. Brewer, Robert Waterston, Christine Queitsch

**Affiliations:** * Department of Genome Sciences,; † Molecular and Cellular Biology Program, University of Washington, Seattle, 98195,; ‡ Department of Molecular Genetics, University of Toronto, Ontario M5S 1A8, Canada, and; § Altius Institute for Biomedical Sciences, Seattle, Washington 98121

**Keywords:** Ribosomal DNA, Repetitive DNA, Whole Genome Sequencing, Copy Number Variation

## Abstract

Individuals within a species can exhibit vast variation in copy number of repetitive DNA elements. This variation may contribute to complex traits such as lifespan and disease, yet it is only infrequently considered in genotype-phenotype associations. Although the possible importance of copy number variation is widely recognized, accurate copy number quantification remains challenging. Here, we assess the technical reproducibility of several major methods for copy number estimation as they apply to the large repetitive ribosomal DNA array (rDNA). rDNA encodes the ribosomal RNAs and exists as a tandem gene array in all eukaryotes. Repeat units of rDNA are kilobases in size, often with several hundred units comprising the array, making rDNA particularly intractable to common quantification techniques. We evaluate pulsed-field gel electrophoresis, droplet digital PCR, and Nextera-based whole genome sequencing as approaches to copy number estimation, comparing techniques across model organisms and spanning wide ranges of copy numbers. Nextera-based whole genome sequencing, though commonly used in recent literature, produced high error. We explore possible causes for this error and provide recommendations for best practices in rDNA copy number estimation. We present a resource of high-confidence rDNA copy number estimates for a set of *S. cerevisiae* and *C. elegans* strains for future use. We furthermore explore the possibility for FISH-based copy number estimation, an alternative that could potentially characterize copy number on a cellular level.

Efforts to understand the genetic basis of complex traits and diseases have almost exclusively focused on the role of single nucleotide variants (Press *et al.* 2019). However, vast genomic variation exists beyond the single nucleotide level, in the form of short tandem repeats, long repetitive regions, and transposable elements (Press *et al.* 2019). This variation remains poorly characterized, not just in humans but even in the most tractable model organisms. The copy number of repetitive DNA elements changes frequently through expansion and contraction, and as a consequence linkage of specific repeat numbers to surrounding single nucleotide variation is markedly reduced (Willems *et al.* 2014; Rabanal *et al.* 2017b, 2017a; Press *et al.* 2018). Hence, the power to use nearby common single nucleotide variants to tag repetitive DNA genotypes in genome-wide association studies is limited (Press *et al.* 2018; Fotsing *et al.* 2019).

Recently, long-read technology, such as PacBio and Oxford Nanopore, has made accessible some forms of repetitive DNA, including complex tandem repeats and short tandem repeats (Ummat and Bashir 2014; Yoshimura *et al.* 2019), although both technologies are plagued by high error rates (Rhoads and Au 2015; Lower *et al.* 2018; Tyler *et al.* 2018). Short tandem repeats (units of 2-10 bases) can also be genotyped with repeat-spanning sequence reads through a capture-based method (Carlson *et al.* 2015; Press *et al.* 2018) or sophisticated computational tools (Willems *et al.* 2017; Bakhtiari *et al.* 2018; Saini *et al.* 2018; Matyášek *et al.* 2019). Copy number variation of short tandem repeats can have significant impact on phenotype; examples include the well-known polyglutamine expansion disorders such as Huntington’s in humans, various examples of incompatibility among closely related species or ecotypes, and altered environmental responses and other complex traits (Undurraga *et al.* 2012; Press *et al.* 2014, 2018). In humans, variation in short tandem repeat copy number in promoter and enhancer regions is implicated in gene expression variation in ∼3000 loci, some of which may drive signals in previously published GWAS studies for height and schizophrenia (Quilez *et al.* 2016; Gymrek *et al.* 2016; Fotsing *et al.* 2019).

Given that short tandem repeats are just one class of repetitive DNA that is ignored in current genotype-to-phenotype association, the characterization of repetitive DNA elements is clearly a critical step in fully understanding human health and disease (Eichler *et al.* 2010). However, despite advances in sequencing technology, some types of repetitive DNA remain recalcitrant to genotyping, due to the extreme length of their repeat units, which can consist of tens of kilobases. For example, PacBio sequencing of *Caenorhabditis elegans* genomes failed to determine repeat copy numbers for several repetitive DNA loci, among them the ribosomal RNA genes, or rDNA (Yoshimura *et al.* 2019).

Nearly all eukaryotes maintain their rRNA genes in large tandemly-repeated arrays. For the majority of plants and animals, the two different pre-rRNA transcripts (the large PolI transcript, variably referred to as 45S or 37S and the small PolIII transcript referred to as 5S) are present in separate array repeats. The PolI transcript is processed into the 18S, 5.8S, and 26S rRNA species (the 26S sometimes termed 25S or 28S). A single repeat unit is 9.1kb in *Saccharomyces cerevisiae*, 7.2kb in *C. elegans* and 43kb in humans, meaning that rDNA, in the most extreme reported cases, may represent up to 15%, 2.8%, or 1% of these genomes (Henderson *et al.* 1972; James *et al.* 2009; Thompson *et al.* 2013). In humans, the short arms of the five acrocentric chromosomes (13, 14, 15, 21, and 22) are almost entirely rDNA (Henderson *et al.* 1972).

Despite the fact that rDNA arrays contribute substantially to genomic content, most reference genomes include only a single repeat unit of the rDNA. The actual number of repeats is remarkably variable (Rogers and Bendich 1987; Lyckegaard and Clark 1989; Thompson *et al.* 2013; Gibbons *et al.* 2014; Bughio and Maggert 2019; Salim and Gerton 2019). rDNA copy number among natural isolates of the same species frequently varies as much as 10-fold; in *S. cerevisiae* reported numbers range from 54 to 511 copies (per haploid genome) (James *et al.* 2009), in maize, from 1,061 to 17,347 copies (per haploid genome) (Li *et al.* 2018), and in humans 61 to 1,590 copies (per diploid genome) (Parks *et al.* 2018). rDNA copy number variation in *Arabidopsis thaliana* largely accounts for the variation in genome size among strains (Long *et al.* 2013).

The few studies that have examined rDNA copy number variation and phenotype have reported a relationship with genome-wide gene expression and mitochondrial abundance in human samples (Gibbons *et al.* 2014) and a relationship with both gene expression and position effect variegation in *Drosophila* (Paredes and Maggert 2009; Paredes *et al.* 2011). Recent studies suggest a relationship between rDNA copy number loss and cancer (Xu *et al.* 2017; Wang and Lemos 2017; Udugama *et al.* 2018). Ultimately, however, full understanding of any relationship between rDNA copy number and health and phenotype is hostage to our ability to accurately quantify that copy number.

Herein, we compare major copy number estimation methods available for rDNA as an example for other tandemly repeated loci of large (kilobase-scale) units such as satellite DNA (Lower *et al.* 2018). These approaches include pulsed-field gel electrophoresis, droplet digital PCR (ddPCR), and whole genome sequencing (WGS). Of these, pulsed-field gel electrophoresis using a contour-clamped homogenous electric field (CHEF), followed by rDNA-specific Southern blotting and hybridization, remains a gold standard for rDNA genotyping. The directional switching of the electric field resolves DNA bands in the megabase range, allowing for direct comparison of array size to standardized ladders. However, the method is laborious and the sheer size and multi-locus structure of the rDNA in organisms such as humans limits the utility of this technique (Stults *et al.* 2008). Of PCR-based approaches, quantitative real-time PCR (qPCR) remains a popular method (Paredes and Maggert 2009; Hallgren *et al.* 2014; Lu *et al.* 2018; Li *et al.* 2018; Valori *et al.* 2019), albeit only capable of detecting large changes (Weaver *et al.* 2010). Recently, ddPCR has gained attention as a more precise alternative (Hindson *et al.* 2013; Xu *et al.* 2017; Salim *et al.* 2017), offering an estimation of the absolute number of starting target molecules in the reaction. WGS is commonly used to estimate copy number through reads aligned to the repetitive region relative to the rest of the genome (Thompson *et al.* 2013; Gibbons *et al.* 2014, 2015; Xu *et al.* 2017; Wang and Lemos 2017; Parks *et al.* 2018; Li *et al.* 2018). While WGS accuracy in single nucleotide polymorphism detection has been extensively assessed (Glenn 2011; Guo *et al.* 2012), its accuracy for application in large repeat copy number estimation has not been evaluated.

To assess the reliability and reproducibility of these methods, we use a series of test strains of different rDNA copy numbers, in two different model organisms: *C. elegans* and *S. cerevisiae*. These test strains belong to larger collections for which variation in rDNA copy number among strains has been previously reported (Thompson *et al.* 2013; Peter *et al.* 2018). We find that the CHEF and ddPCR methods can provide reproducible copy number estimates; however, the two methods do not yield the same absolute copy number estimates. In contrast, WGS and a novel capture-based method that we attempted to implement produce high error in copy number estimation even for technical replicates. As a future alternative to these techniques, we present the foundation for FISH-based copy number estimation technology.

## Materials and Methods

### Strains and sample collection

#### C. elegans:

Wild isolates of *C. elegans* were kindly provided by the Moerman lab (strains MY1, PX174, JU775, MY16, MY14, MY6, ED3040, and ED3042). The eight wild isolate *C. elegans* strains were grown to starvation on 15cm high-peptone (20g/L) NGM NA22 plates, enriching for arrested L1s. These plates were washed with 15mL M9, centrifuged 450xg 2min. Supernatant was removed and the pellet was resuspended in 8mL water and spun again 450xg 2min. Approximately 75μL of worm pellet was put into a 1.5mL tube to which 200μL ATL buffer was added (Qiagen DNeasy kit 69504) before putting the tube at -20° for eventual genomic DNA extraction and whole genome sequencing (see below). 80μL of the same pellet was embedded in agarose plugs for CHEF gel application (see below).

#### S. cerevisiae:

Strains from the “1,011 *S. cerevisiae* isolates” collection (Peter *et al.* 2018) were generously provided by the Dunham Lab. The laboratory control strain BY4741 and the strain with 35 rDNA copies were obtained from the Brewer Lab. Strains were streaked out on YPD plates and incubated for 2 days at 30°. Cells from the patch population and 3 individual colonies were then separately inoculated in 5mL synthetic complete liquid media buffered with succinic acid and grown for 2 days at 30°. Cells were then collected into 1.5mL tubes, pelleted, washed once with 50mM EDTA, and stored as dry pellets at -20° until either preparation of CHEF gel plugs or genomic DNA extraction.

### CHEF gel sample preparation and run conditions

#### C. elegans:

720μL of melted 42° 0.5% SeaPlaque GTG agarose was added to an 80μL worm pellet described above. ∼80μL of this suspension was placed in agarose plug molds (Bio-Rad #1703713), on ice, and allowed to solidify at 4° for at least 30min. Plugs were extracted into 2mL tubes, to which was added 300μL TEL [9mM Tris, 90mM EDTA pH 8, 10mM levamisole], followed by incubation on ice 30min. The levamisole was found to be necessary to prevent the worms from crawling out of the plug during lysis. The supernatant was removed from the tube, being careful to avoid damaging the plugs, and replaced with 300μL lysis buffer [1% SDS, 1mg/mL Proteinase K (Sigma-Aldrich P4850), 8mM Tris, 80mM EDTA pH 8, 1mM levamisole]. Tubes were put at 50° for ∼24 hr to allow lysis of the worms. L1 stage worms are reported to have a weak enough cuticle for in-plug digestion. After digest, plugs were decanted into 24-well plates (two plugs per well). Supernatant was removed, the plugs were rinsed once with 300μL TE [10mM Tris, 1mM EDTA], new TE was added and the plugs were rocked at room temperature 2hr. Supernatant was removed and replaced with fresh TE and rocked overnight at 4°. In total, eight of these TE washes were performed, with at least one taking place at 4° overnight. After the last wash, plates were stored in TE and placed at 4° until use.

Plugs were prepared for CHEF gel by digestion with an enzyme that cuts in chromosome I but not within the rDNA. We found SwaI to be the best restriction enzyme for this purpose; SwaI cuts 3927bp upstream of *rrn-3.56*. Plugs were soaked in 1X NEB 3.1 buffer for 30min, buffer was replaced with fresh 1X NEB 3.1, and plugs were soaked another 1hr. This incubation was done without rocking, with the plate on ice. Plugs were then transferred out of buffer to a parafilm-wrapped slide and 4μL of SwaI was added to the top of the plug. The plugs were placed in a container with a wet paper towel and incubated at 25° for 4hr before CHEF gel loading. To load, plugs were transferred to the teeth of a gel comb, and 1% agarose in 0.5X TBE was poured around them, careful not to dislodge. Once the gel solidified, it was placed in a CHEF gel box (Bio-Rad CHEF DRII), where the gel was run at 100V for 68hr, 14°, switch times = 300-900 sec. Replicates 2 and 3 were digested with SwaI, but replicate 1 used MfeI (digests 1057bp upstream of *rrn-3.56*); we found MfeI to have more off-target cutting (more degradation of rDNA) than SwaI. Ladders of two different ranges were included: *S. cerevisiae* chromosomes were used as one ladder (maximum size 2.5 Mb), and *Hansenula wingei* chromosomes as another ladder (maximum size 3.13 Mb, Bio-Rad 170-3667). After the gel had been run, it was soaked in ethidium bromide (0.3μg/mL in 0.5X TBE) to visualize the ladders.

See Supplemental Data for Southern blotting and probe conditions.

#### S. cerevisiae:

*S. cerevisiae* genomic DNA plugs were prepared as previously published (Tsuchiyama *et al.* 2013). 1% low-melt agarose (Lonza SeaPlaque GTG agarose) in 50mM EDTA was melted and cooled in a 45° water bath for 10 min. Approximately 10^8^ cells (0.5mL of 2-day culture, frozen), were transferred to a 1.5mL tube and resuspended in 100μL 50mL EDTA pH 8.0. Cells were then mixed with 100μL 1% low-melt agarose, transferred to agarose plug molds (Bio-Rad #1703713, 2 plugs generated for each sample), and incubated at 4° for 15 min to solidify agarose. Plugs from a single strain were then transferred to a single well in a 24-well plate containing 1mL spheroplasting solution (1M Sorbitol, 20mM EDTA, 10mM Tris-HCl pH 7.5, 14mM β-mercaptoethanol, 0.5mg/mL Zymolyase 20-T), and incubated for 4 hr in a 37° incubator with periodic agitation. Spheroplasting solution was then removed, plugs were washed 1x15 min with 1mL LDS solution (1% lithium dodecyl sulfate, 100mM EDTA, 10mM Tris-HCl, pH 8.0), and then incubated overnight in a 37° incubator with 1mL fresh LDS solution. In the morning, plugs were washed 3 times for 20 min with 0.2X NDS solution (1X NDS: 0.5M EDTA, 10mM Tris base, 1% Sarkosyl, pH 9.5), and then 5+ times with TE pH 8.0. Plugs were then stored in TE pH 8.0 until use.

For CHEF gels examining the sizes of Chromosome XII, which contains the rDNA: 200mL of 0.8% LE agarose in 0.5X TBE was melted and then cooled in a 50° water bath for 10 min. A slice of an agarose plug (∼2mm) from each strain was transferred to a separate tooth on a gel-comb and excess moisture was wicked away using a Kimwipe. An *H. wingei* standard ladder (Bio-Rad 170-3667) sample was included on the comb on a separate tooth. The gel-comb with plug slices was then positioned in a Bio-Rad CHEF gel-casting tray and embedded in the prepared 0.8% LE agarose. Once the gel was solidified, the gel-comb was removed and the gel was then transferred to a CHEF gel module (Bio-Rad CHEF-DRII) containing 2.3L 0.5X TBE continuously cooled to 14°. Yeast Chromosome XII CHEF gels were run for 66 hr at 100V, switch times = 300-900 sec.

For CHEF gels examining the size of *S. cerevisiae* rDNA arrays excised from Chromosome XII: The *S. cerevisiae* rDNA array contains no BamHI restriction sites; the nearest BamHI sites are 8.8kb from the centromere proximal edge and 30.9kb from the telomere proximal edge. To digest the rDNA array away from Chromosome XII, agarose plugs were washed for 20 min three times in 1mL 1X NEB Buffer 4 + 1X BSA. Two 2mm slices from an agarose plug were then transferred to a dry 24-well plate, plug slices covered with 50μL BamHI-HF restriction digest reaction (1X NEB Buffer 4, 1X BSA, 1.3μL BamHI-HF), and incubated in a 37° incubator for 5 hr. 1mL TE pH 8.0 was then added to each well to facilitate plug slice handling. Each of the 2 plug slices were run on two separate CHEF gels: the “High Molecular Weight” protocol described above (100V, 66 hr, 300-900 sec), which resolves fragments between 1Mb-3.13 Mb, and the “Low Molecular Weight”(165V, 66 hr, 47-170 sec), which resolves fragments between 225kb-1.1Mb. Both BamHI-digested sample CHEF gels were cast as described above for the uncut CHEF gel in 0.8% LE agarose with the appropriate standard ladder: the Bio-Rad *H. wingei* for the “High Molecular Weight” CHEF gel run conditions and the NEB Yeast Chromosome PFG Marker for the “yeast full chromosome” run.

Southern blotting was performed as described for the *C. elegans* samples (see Supplemental Data), using yeast-specific probes. For the uncut CHEF gels, a single copy sequence on Chromosome XII (*CDC45*) was used as a probe for Chromosome XII location. For BamHI-digested CHEF gel samples, the *S. cerevisiae* rDNA NTS2 sequence was used as a probe for the location of the excised rDNA array.

### Genomic DNA extraction and whole genome sequencing sample preparation

#### C. elegans:

The Qiagen DNeasy kit (69504) was used for worm genomic DNA extraction. Wild isolate worm pellets frozen in ATL were freeze-thawed three times between -20° and 37°. 20μL proteinase K was added and the samples were incubated at 56° for 3hr with occasional vortexing. 4μL 100mg/mL RNase A (Qiagen 19101) was added to each sample and incubated at room temperature 5min. 200μL AL buffer was added, and the DNA extraction continued as described in the kit protocol. Final DNA was eluted in a total volume of 150μL. For each worm wild isolate, a single genomic DNA sample was stored at 4° and used for all of the described library preparations for that strain. The dates of each library preparation are provided in Supplemental Material, Table S1.

Sequencing of libraries was performed using an Illumina Nextera DNA Sample Preparation kit (FC-121-1030). All worm library preparations were performed by the same person. For 10ng input: 10ng of input gDNA was brought up to 9μL with water. 10μL tagmentation buffer and 1μL tagmentation enzyme were added and mixed by pipetting up and down. Samples were incubated 55° for 8min, then the reaction was halted by addition of 10μL 5M guanidine thiocyanate, mixed by pipetting, and incubated at room temperature for 3min. AMPure XP beads (Beckman Coulter A63881) were used for DNA purification: 15μL AMPure beads and 25μL binding buffer [20% PEG8000, 2.5M NaCl] were added to the reaction and mixed by pipetting. The reaction was allowed to sit at room temperature 10min before being placed on a magnet stand to attract the beads. After >2min on the magnet, the supernatant was removed from the beads and 150μL 70% ethanol was immediately added, allowed to sit for >30sec, removed, and another 150μL 70% ethanol added. After >30sec, the ethanol was removed completely and the bead pellet was allowed to dry ∼30sec. The tubes were removed from the magnet stand and the pellet was resuspended in 20μL elution buffer (Qiagen EB 1014608). After 2min incubation at room temperature off of the magnet, the tubes were returned to the magnet for >2min, after which the DNA-containing liquid was transferred to a new tube. The 50ng input library preparations were performed similarly, with the following volume adjustments: 50ng of gDNA was brought up to 20μL volume with water, 25μL tagmentation buffer was used, 5μL tagmentation enzyme, 25μL 5M guanidine thiocyanate, 20μL AMPure beads, and 80μL binding buffer. Wash and elution volumes were the same.

PCR amplification of the libraries was done using Illumina NPM master mix (part of kit FC-121-1030). 10μL of the above tagmented DNA was put into each reaction, along with NPM and Illumina barcode index primers (FC-121-1012). Dual barcoding was used. PCR conditions were 72° 3min, 98° 30sec, 98° 10sec, 63° 30sec, 72° 40sec, with these latter three steps cycled 6 times. For trials 1 and 2 of both input amounts, SYBR green dye (Thermo Fisher S7563) was also added to the reaction, to visually assess amplification. Post-PCR, libraries were again purified by addition of 30μL AMPure XP beads to the PCR reaction, incubation at room temperature for 5min, incubation on the magnet for 2min, liquid removal and addition of 200μL 80% ethanol to wash followed by a second wash of the same, removal of all ethanol, letting dry for 30sec, removal from magnet and resuspension of bead pellet in 32.5μL resuspension buffer (Illumina FC-121-1030), incubation at room temperature 2min, incubation on the magnet >2min, and recovery of purified suspended DNA into a new tube.

Final libraries were quantified by Qubit high sensitivity assessment (Invitrogen Q32854) and diluted to 2nM. Denaturation and dilution of libraries for sequencing was done as described (NextSeq Denature and Dilute Libraries Guide 15048776 Rev. D). Sequencing was done using 75bp-paired end NextSeq 500/550 High Output v2 150 Cycle kits (FC-404-2002).

#### S. cerevisiae:

*S. cerevisiae* genomic DNA was extracted using a phenol:chloroform “Smash and grab” protocol (Rose 1990). To the 1.5mL tube containing the frozen pellet of ∼3 × 10^8^ cells (1.5mL of 2-day culture), we added 0.1mL of acid-washed glass beads, 0.2mL of lysis buffer (10 mM Tris, pH 8.0, 1 mM EDTA, 100 mM NaCl, 1% SDS, 2% Triton X-100), and 0.2mL of 25:24:1 phenol:chloroform:isoamyl alcohol and vortexed for 2min. 0.2mL of TE (10mM Tris, 1mM EDTA, pH 8.0) was added, the tube was inverted to mix and centrifuged at 14,000 rpm for 5 min to separate the phases. The DNA-containing aqueous phase was transferred to a new 1.5mL tube containing 1mL 0.5M potassium acetate in 100% EtOH and centrifuged at 14,000 rpm for 5 min to precipitate the DNA. DNA was resuspended in 150μL 10mM Tris pH 8.0 + 0.1ng/mL RNase A, incubated at 37° for 20 min to degrade RNA. To each RNaseA-treated sample, 150μL phenol:choloroform:isoamyl (25:24:1) was then added, vortexed, and centrifuged again at 14,000 rpm for 5 min, aqueous phase transferred to a new tube. For ddPCR and sequencing library generation, 50μL of the DNA-containing aqueous phase was further purified using a Zymo Research Clean & Concentrator column (D4013) as per manufacturer instructions and resuspended in 50μL 10mM Tris pH 8.0. Libraries were prepared on 10ng yeast gDNA as for worms (above) with the exception that library amplification was done using Kapa HiFi Readymix (Kapa Biosystems KK2602).

### Relative read coverage-based rDNA copy number estimation

#### C. elegans:

Methods were modeled after (Thompson *et al.* 2013). Reads were demultiplexed from the NextSeq and FASTQ files were aligned to the unmasked WS235 genome with bowtie2/2.2.3 to generate .sam and .bam files (Langmead and Salzberg 2012; Langmead *et al.* 2019). Reads mapping to multiple locations were randomly assigned to one. A custom Perl script (Thompson *et al.* 2013) was used to count the total number of mapped reads in the .bam, and the total number of reads mapping to the rDNA coordinates, using samtools/0.1.18 (Li *et al.* 2009). rDNA coordinates (including *rrn-3.56* and *rrn-1.2*) used for WS235 were ChrI 15060299-15071033. Copy number of rDNA was calculated by the ratio of these two counts, corrected for the length of the rDNA (7197bp) and the length of the genome (100286070bp), with the equation:

$$\left({\text{rDNA\_counts}}^{*}\text{100286070}\right)/\left({\text{total\_counts}}^{*}\text{7197}\right)=\text{rDNA copy number}$$

Each line of the bam, and thus each end of a paired end read, was counted independently. Read duplication removal was not used because the repetitive nature of the rDNA engenders a situation in which reads with identical starts and ends nevertheless represent true independently-generated reads and should not be removed.

See Supplemental Data for further analysis description.

#### S. cerevisiae:

Methods were modeled after (Thompson *et al.* 2013) and adapted for the *S. cerevisiae* genome. FASTQ files were downloaded from SRA with wget and split into forward and reverse paired read files. SRR numbers are indicated in Table S7. For in-house sequencing, reads were split as above. Split, paired FASTQ files were aligned to the unmasked *S. cerevisiae* S288C R64 genome. A custom Perl script was used to count the total number of mapped reads and the total number of rDNA-mapping reads.

See Supplemental Data for further analysis description.

### FISH sample preparation

For six wild isolate *C. elegans* strains (JU775, ED3042, ED3040, MY6, PX174, and MY1), ∼40,000 starved L1s were plated on a 15cm high-peptone (20g/L) NGM plate with NA22 and grown five days at 16° to reach gravid adulthood. Worms were washed from the plates with 50mL sterile M9. Adults were allowed to settle to the bottom of the tube for 3min, after which most of the M9 was aspirated, and new M9 was added. Three washes total were performed, before adding 25mL bleach solution to the worms [0.5M KOH, 10% BDH sodium hypochlorite solution (BDH7038)]. Worms were rocked in bleach solution 5min followed by 20sec vortexing, then addition of M9 up to 50mL. Embryos were centrifuged 1500rmp for 1min, resuspended in 50mL sterile egg buffer [118mM NaCl, 48mM KCl, 2mM CaCl_2_•2H_2_O, 2mM MgCl_2_•6H_2_O, 25mM Hepes, pH 7.3] and centrifuged 1500rpm for 2min. The supernatant was aspirated and the pellet was resuspended in 10mL egg buffer. 5mL of egg suspension was added to two 15mL tubes containing 5mL 60% sucrose (for final concentration of 30% sucrose). Tubes were vortexed and then centrifuged 200xg for 3min to separate embryos from debris. ∼1mL per tube of the white egg layer was moved into a new 50mL tube. The embryo suspension was brought up to 40mL with egg buffer, spun for 2min at 1500rpm, then washed again with another 40mL egg buffer and spun again. The final volume was brought down to ∼1mL. To this suspension was added 2mL of 1U/mL chitinase (Sigma-Aldrich C6137) and the tube was let sit at room temperature 30 min to digest the egg shell. The solution was then split into two 1.5mL tubes and 100μL pronase (Sigma-Aldrich P6911) was added. A 21 gauge needle was used to draw the embryos up and down 40 times, with a 5min room temperature incubation half-way through. The tubes were spun 1300xg 1min before taking the supernatant off and combing the two cell pellets. Three more washes of 1mL egg buffer and 1300xg 1min spins were performed before final resuspension in 500μL egg buffer. Cells were temporarily stored on ice until preparation for FISH, done the same day.

Cells were filtered using a 100μm cell strainer and resuspended in 1x PBS at a density of 10^6^ cells per ml. About 100 ul of cell solution was seeded on Poly-L-Lysine (Sigma, P1399) coated cover slips (1.5, 18x18mm, Fisher Scientific) and air dried for 10 min at room temperature for cells to adhere to coverglass. Cells were then fixed with 4% Paraformaldehyde (Polysciences, 18814-10) in 1x PBS for 10 min at room temperature and subsequently washed 3x with PBS (5 min each) to clear the fixative. Cells were then permeabilized for 10 min with 0.5% Triton X-100 in PBS and 0.1M HCl for 5 min, with intermediate PBS washes (3x5 min). After 2x5 min wash with 2x Sodium Citrate (SSC), cells were incubated with RNaseA (25 ug/ml) for half hour at 37°, followed by denaturation in 50% formamide for another half hour at room temperature and 70% formamide for approximately 5 min at ∼78°. Cells were incubated at 37° overnight with Quaser-670 labeled oligo-FISH probes that were designed for the same genomic locations that corresponded to smMIPs. Post hybridization, cells were washed 2x with 0.2% Tween in SSC, counterstained with DAPI for 10 min, and then mounted onto glass slides with Prolong Gold antifade. Stained cells were imaged in 3D using a conventional widefield microscope (Nikon Ti) fitted with an Andor Zyla 4.2CL10 CMOS camera (pixel size = 6.5μm). Acquired 3D images were processed using Matlab (v2016b, Mathworks, Natick MA) scripts to delineate individual cell nuclei and rDNA FISH foci, and additionally, estimate the size and intensity of each FISH spot.

### Droplet digital PCR

*S. cerevisiae* genomic DNA was diluted to 0.05 ng/ul in low-bind tubes. Each 20µl reaction consisted of 10µl 2X ddPCR Supermix for Probes (Bio-rad), 0.125µl EcoRI-HF (NEB, 20,000 U/mL), 1.8µl of 10µM Primer Mix (containing 10µM each rDNA F and R primers and Tub1 F and R primers), 1µl of 5µM Probe mix (containing 5µM each rDNA and Tub1 probes), and 1µl of DNA at 0.005 ng/µl. The mixture was incubated for 15 min for DNA digestion to occur, followed by droplet generation on a QX200 Droplet Generator (Bio-rad). Amplification was performed for 50 cycles with a 57° annealing temperature, and droplet reading was performed on a Bio-rad QX200 Droplet Reader. Optimal annealing temperature was determined by a temperature gradient of 56-62° with BY4741 DNA (Fig S2).

Additional materials and methods are available in Supplemental Data.

### Data availability

FASTQs of whole genome sequencing are available at SRA (PRJNA565452 for *C. elegans* data, PRJNA573925 for *S. cerevisiae* data). Supplemental Data including supplemental methods, figures, and tables are available at figshare: https://doi.org/10.25387/g3.10406519.

## Results

### Whole genome sequencing copy number calls are error-prone

**Figure 1:**
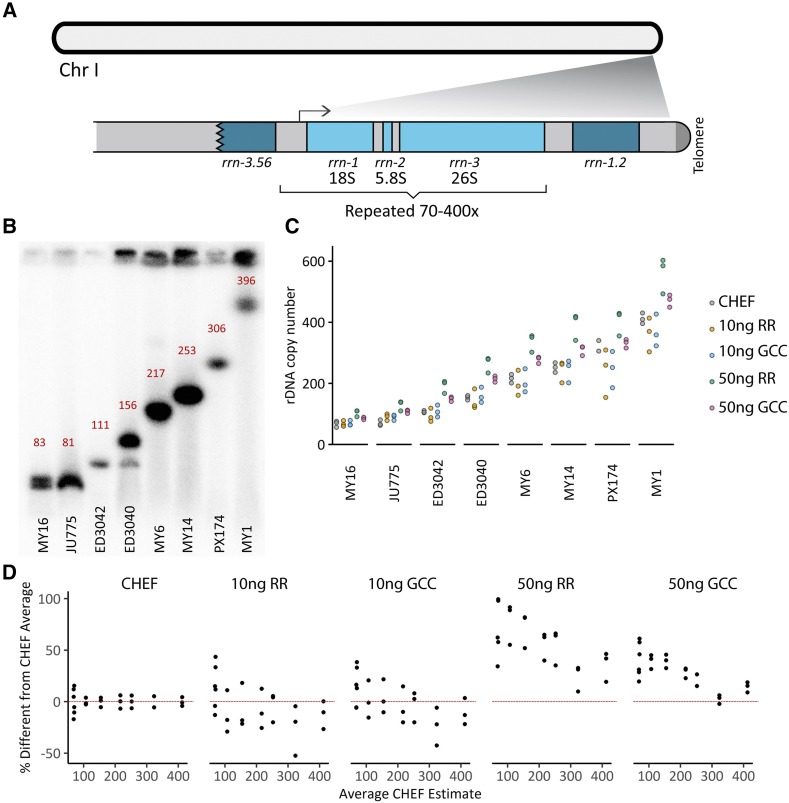
(A) Schematic of the *C. elegans* rDNA locus, at the right arm of chromosome I. The 18S, 5.8S, and 26S rRNAs are transcribed as one unit and later processed into the three species. One repeat unit is 7.2kb in length and is tandemly repeated approximately 70 to 400 times, depending on the strain. The array is flanked by a partial copy of the 26S rRNA gene (*rrn-3.56*) and an additional copy of the 18S (*rrn-1.2*), which ends approximately 1kb upstream of the telomere. (B) Southern blot against rDNA from a CHEF (contour-clamped homogenous electric field) gel reveals rDNA copy number for eight wild isolates of *C. elegans*. Band size was measured relative to yeast chromosomal ladders visualized by ethidium bromide staining (Fig. S1), and copy numbers calculated from the band size for each primary band are listed in red (also in Table 1). MY16 displays two bands of similar intensity, copy numbers 83 and 69; only the upper number is listed in the image. (C) rDNA copy number (per haploid genome) was estimated for eight *C. elegans* wild isolates in five different ways: CHEF gel followed by Southern blot, 10ng input Nextera-based whole genome sequencing followed by relative read count coverage of the rDNA without or with GC content correction (“RR” and “GCC,” respectively), and 50ng input whole genome sequencing with RR and GCC. Three replicates for each are plotted. (D) Data from (C) is plotted as a percentage of the average CHEF-based values ((estimated number – CHEF number)/CHEF number)). The red line indicates 0% error.

In *C. elegans*, the 18S, 5.8S, and 26S rRNA genes are tandemly repeated at a single locus (45S locus) on the end of the right arm of chromosome I (Figure 1A). Forty *C. elegans* wild isolates were recently sequenced to high depth using short-read sequencing (Thompson *et al.* 2013). The reported rDNA copy number estimates vary from 68 to 418 per single copy of the genome (*i.e.*, haploid number)(Thompson *et al.* 2013). We wanted to examine the accuracy of these estimates, using WGS and CHEF gel analysis to estimate rDNA copy number in eight of these lines spanning the range of reported values: 68, 70, 105, 149, 193, 237, 298, and 418 (Table 1). From a single large plate of each worm strain, we collected a population of worms and split them into tubes for genomic DNA extraction and for CHEF gel analysis, to compare these methods across identical biological samples.

**Table 1:** rDNA copy number estimates of *C. elegans* strains using CHEF gel or WGS

		CHEF gel				Nextera Whole Genome Sequencing											
						10ng input						50ng input					
Strain	WGS*^a^*	Rep1	Rep2	Rep3	Ave	RR*^b^*	GCC*^c^*	RR	GCC	RR	GCC	RR	GCC	RR	GCC	RR	GCC
MY16	68	56	76*^d^*	71*^d^*	**68**	65	64	60	64	78	79	110	87	110	89	91	81
JU775	70	63	81	66	**70**	100	93	78	79	93	96	139	112	138	110	110	102
ED3042	105	104	111	106	**107**	88	106	76	90	119	129	206	155	202	151	167	141
ED3040	149	146	156	160	**154**	121	154	127	138	182	187	281	224	279	216	234	204
MY6	193	201	217	229	**216**	191	194	161	173	243	248	356	285	351	283	302	265
MY14	237	236	253	267	**252**	266	259	202	202	263	273	419	319	415	319	341	291
PX174	298	—	306	340	**323**	260	252	154	186	309	304	429	343	425	335	355	316
MY1	418	410	396	431	**412**	370	359	303	323	414	427	603	489	585	475	493	449

a rDNA copy number reported by WGS with sonication and adapter ligation in Thompson *et al.* 2013, *Genome Res*.

b Relative read count coverage based rDNA estimate.

c GC-content corrected rDNA estimate.

d Average of two bands.

For CHEF gel analysis, we embedded worms in agar before proteinase digestion to preserve chromosome integrity. A restriction enzyme was used to digest most of chromosome I, leaving only an intact rDNA array. We ran the genomic fragments on a CHEF gel along with yeast chromosome ladders as size markers and then Southern blotted and hybridized with an rDNA probe (Figure 1B). Copy number per genome was calculated by dividing the total size of the array by the known size of single repeat unit (7.2kb). Replicating the CHEF gel results thrice revealed high reproducibility in copy number determination (Table 1). A strain with reported rDNA size of 418 copies (MY1) ranged in copy number from 396 to 431, and a strain with reported rDNA size of 70 copies (JU775) ranged in copy number from 63 to 81. Among all strains, the variation in copy number estimates within technical replicates ranged from 3% CV to 15%, with a median of 6% (CV=(standard deviation/mean)*100).

Notably, CHEF gel analysis compares samples to DNA molecules of known size, something other techniques lack, providing confidence in the resulting copy number estimates. Therefore, for the subsequent troubleshooting and quality assessments, we use the average value of the three replicate CHEF gels as the ‘true’ value of rDNA copy number in our samples and gauge our accuracy with respect to these values. Of additional note is that the CHEF approach also reveals minor bands, if present, possibly representing copy number differences in a subset of the population (Figure 1B, ED3040 lane). Pooled amplification measurements reflect only average rDNA array size, therefore information about the potential existence of subpopulations or heterozygosity is lost in sequencing or PCR-based approaches.

To assess the accuracy of WGS with respect to CHEF gels, we used genomic DNA extracted from the worm samples to generate barcoded libraries by Nextera tagmentation and amplification. This method differed from the published study (Thompson *et al.* 2013), which used sonication and adapter ligation. Two different DNA input amounts were tested: 10ng and 50ng genomic DNA. Three independent replicate libraries of each input amount were prepared from the same tube of genomic DNA. We sequenced these libraries with an Illumina NextSeq 500 and used read coverage of rDNA sequence relative to the whole genome to estimate rDNA copy number, an approach used elsewhere (Thompson *et al.* 2013; Gibbons *et al.* 2014; Xu *et al.* 2017; Wang and Lemos 2017).

By this initial estimation method, we observed surprising variability in the rDNA copy number calculated for each library, despite their identical source material. Libraries of the strain JU775 (CHEF-based copy number of 70) reported copy numbers from 78 to 139. Libraries from the strain MY1 (CHEF-based copy number of 412) reported copy numbers from 303 to 603, a much less precise range than that produced by CHEF gel (Table 1, Figure 1C). The 50ng input DNA samples were particularly distorted, with the percent variation from the CHEF estimate as much as 100% (calculated as 100 * absolute value of (estimated number – CHEF number)/CHEF number)). Even the 10ng input DNA samples varied from 0.6 to 52% (Table S1). On average, the 10ng and 50ng input amounts varied 18% and 58% in their copy number estimates, respectively (Table S2), an unacceptably high error for many applications.

### Computational correction can improve WGS-based copy number estimates

We asked what computational measures could be taken to improve accuracy of the WGS data. We examined factors including GC content bias, total read coverage, input amount, and single copy region coverage (Table S1).

First, we attempted to correct for tagmentation enzyme bias by implementing a maximum likelihood estimation GC-content correction method (Benjamini and Speed 2012; Parks *et al.* 2018). This approach corrects for GC content bias and read coverage in a sample-specific manner. Implementation of this method improved copy number estimates, but error ranges remained high: 0.2–42% (average 15%) in the case of 10ng input, and 2–61% (average 29%) in the case of 50ng input (Table S1, S2, Figure 1C, D). Of the conditions tested here, lower input amount (10ng) and GC-content correction brought copy number estimates generally closest to CHEF-based values (Table 1, Figure 1D). Although exhibiting greater deviation from the CHEF-based copy number, the combination of higher input amount (50ng) and GC-content correction produced the highest degree of reproducibility (Figure 1D, Table 1). This observation suggests a potential vehicle to computationally correct WGS-based copy number estimates, assuming benchmark samples of CHEF-based copy number have been included. The high reproducibility observed in the 50ng input condition could also represent an artifact of closely spaced library preparation, as two of the three samples under the 50ng input condition were prepared on sequential days (day of library preparation provided in Table S1).

Insufficient read coverage seemed another highly plausible source for inaccuracy of WGS-based copy number estimates. However, we did not observe a correlation between the total reads a sample received and its accuracy in copy number call. Pearson’s correlation between total aligned reads and the error of the WGS-based rDNA estimates was 0.06 and 0.12 for the original and GC-corrected copy number estimates, respectively, both non-significant (Table S1). We furthermore performed an *in silico* downsampling experiment with published data from strains MY1 and JU775 (Thompson *et al.* 2013). Repeated downsampling to a randomly drawn 5% of the initial reads introduced small differences in copy number calls of up to 0.9% or 2.4%, for MY1 and JU775, respectively (Table S3). This amount of variation was far below the variation we see among library preps of comparable coverage, suggesting coverage is not the major contributor to error in copy number calls.

Changes to other WGS processing measures failed to improve copy number estimates. While still employing GC-content correction, we compared WGS-based estimates using different alignment algorithms, to little effect. BWA-MEM alignment gave average error of 15% and 23% for 10ng and 50ng input, respectively. This degree of error compares to the Bowtie 2 alignment used above, with average error of 15% and 29% (Table S4). Similarly, comparing alignment with and without read adapter trimming gave very similar estimates: 15% and 27% average error with adapter trimming compared to 15% and 29% without, for 10ng and 50ng input, respectively (Table S4).

We asked if samples that were miscalling rDNA copy number were also distorted in their copy number estimates of other regions. To do so, we examined twenty-nine 7.2kb regions of the genome, with representatives from all six chromosomes that should be present at single copy (Table S5). We estimated copy number for each of these regions as the rDNA locus copy number was calculated, using read coverage of each region relative to whole genome (Table S6). The average of these estimates for each strain ranged from 0.81 to 1.44 and was correlated with WGS-based rDNA copy number (Pearson’s value of 0.93), indicating that some samples were indeed prone to inflated copy number calls of not just the rDNA locus (Table S1).

### A panel of yeast strains confirms high error in WGS-based copy number estimates

**Figure 2:**
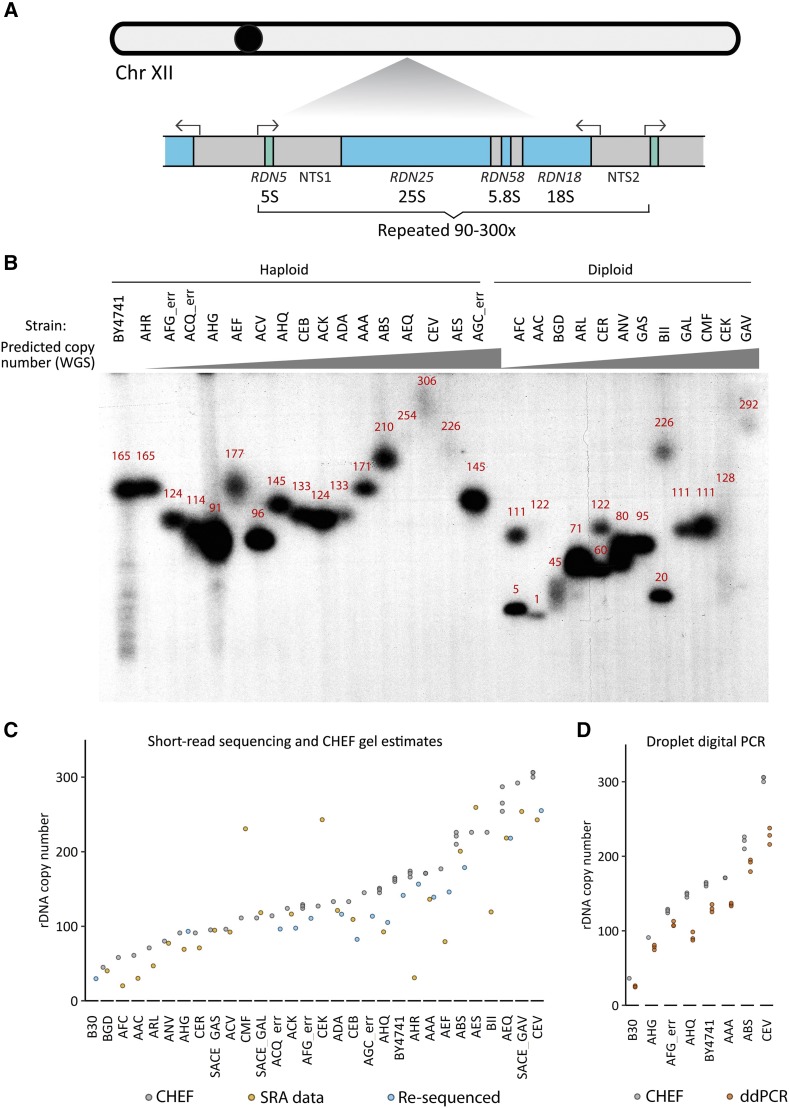
(A) Schematic of the *S. cerevisiae* rDNA locus on chromosome XII. The 18S, 5.8S, and 25S are transcribed as one unit by PolI. 5S rRNA is transcribed as a separate unit by PolIII. The repeating unit is 9.1kb in length and in haploid strains is tandemly repeated approximately 90 to 300 times. (B) Southern blot for chromosome XII from a CHEF gel reveals size variation of *S. cerevisiae* strains from the 1002 genomes project (Peter *et al*. 2018). rDNA copy numbers measured from each band are noted in red, calculated from band size relative to yeast chromosomal ladders. Haploid and diploid strains are noted. Wild haploid yeast strains show copy numbers ranging from 91-306. Wild diploid strains show individual bands with copy numbers ranging from 45-292 (note that two strains, AFC and AAC had bands at the lower limit of our ability to quantify, estimated as 5 and 1 copies, respectively). From left to right, strains were arranged in order of the expected rDNA copy number, based on previously reported whole genome sequencing estimates. (C) rDNA copy numbers for 30 strains of *S. cerevisiae* isolates are plotted, reflecting CHEF-based rDNA copy number estimates(“CHEF”, dark blue), previously reported data (“SRA data”, green), or our own Nextera whole genome sequencing-based estimate (“Re-sequenced”, mauve, done for 14 strains plus B30 and BY4741 controls). The annotation “err” indicates strains whose re-sequenced genotype did not match SRA library genotypes. The SRA-based estimate has therefore been omitted from the graph for these strains. (D) Droplet digital PCR estimations (in triplicate) of rDNA copy number for eight yeast strains are plotted next to their CHEF-based rDNA copy numbers.

The yeast *S. cerevisiae* has arguably the best characterized rDNA locus of any eukaryote (Kobayashi *et al.* 2001; Eickbush and Eickbush 2007; Nomura *et al.* 2013; Woolford and Baserga 2013). Unlike many plants and animals, *S. cerevisiae* contains the genes for all four rRNA species in one array, on chromosome XII (Figure 2A). As in other eukaryotes, this array is highly repetitive, with the laboratory strain BY4741 containing approximately 150 copies of the 9.1 kb repeat unit (Kwan *et al.* 2016). With a tractably-sized genome of ∼12Mb and a single rDNA locus, *S. cerevisiae* presents another excellent choice for WGS-based copy number estimation.

WGS data are available for many wild isolates of *S. cerevisiae*, from which rDNA copy numbers have been reported (Peter *et al.* 2018). We selected a panel of 28 of these strains with reported rDNA copy numbers ranging from 17.5 to 277 per haploid genome (Table 2, Table S7, Table S8)—16 of these were annotated haploid strains and 12 diploid. We quantified rDNA copy number in these strains by CHEF gel analysis as well as by whole genome sequencing on a 14-strain subset of the haploids.

**Table 2:** rDNA copy number estimates of *S. cerevisiae* haploid strains

			CHEF gel				ddPCR		
Strain	SRA Number	WGS*^a^*	Rep1	Rep2	Rep3	Re-sequenced*^b^*	Rep1	Rep2	Rep3
BY4741	—	—	165	160	163	141	126	135	129
B30	—	—				30	25	26	25
AHR	ERR1308770	31	166	174	171	157	—	—	—
AFG_err*^c^*	ERR1309145	50	124	129	127	111	113	107	107
ACQ_err	ERR1308618	74	114	—	—	96	—	—	—
AHG	ERR1308781	69	91	—	—	93	75	78	81
AEF	ERR1308873	79	177	—	—	146	—	—	—
ACV	ERR1308596	92	96	—	—	—	—	—	—
AHQ	ERR1309019	93	145	151	149	105	90	98	87
CEB	ERR1309017	109	133	—	—	82	—	—	—
ACK	ERR1308893	116	124	—	—	97	—	—	—
ADA	ERR1309427	121	133	—	—	116	—	—	—
AAA	ERR1309487	136	171	169	171	139	135	133	137
ABS	ERR1309033	201	210	226	221	179	195	192	180
AEQ	ERR1309512	218	254	287	265	218	—	—	—
CEV	ERR1308745	243	306	306	300	255	238	216	228
AES	ERR1309368	259	226	—	—	—	—	—	—
AGC_err	ERR1309000	275	145	—	—	114	—	—	—

a rDNA copy number estimated from GCC-analyzed WGS data from Peter *et al.* 2018 *Nature*

b rDNA copy number estimated from GCC-analyzed WGS data from this study.

c err notation: re-sequencing data indicates strain genotype did not match SRA data genotype.

CHEF gel analysis revealed copy numbers wildly discrepant from the published WGS-based copy numbers (Figure 2B, C, Fig S1). CHEF-based rDNA copy numbers varied as much as 143 copies and as little as 1 copy from the reported WGS-based numbers (Table 2). As before, we ran triplicate CHEF gels to assess the reproducibility of this technique. Variation in copy number calls among CHEF technical replicates ranged from 0.6% CV to 5.6% (average of 1.8%), consistent with the known high precision of this method.

We furthermore performed CHEF analysis on multiple single colony isolates of the same strain, to examine the possibility that colony-to-colony variability explains differences between WGS- and CHEF-based copy number estimates (Fig S1E). We performed CHEF gel analysis of three separate colony isolates as well as a mixed population for 10 haploid strains and 8 diploid strains. Most strains with discrepant CHEF/WGS estimations displayed no rDNA heterogeneity. Of the four haploid strains that did exhibit different rDNA copy numbers between colonies, two strains had a single colony whose CHEF-based copy number estimate was more similar to the WGS. For the other two strains, the “population” CHEF-based copy number estimate was in agreement with the WGS-based estimate. Although we did not comprehensively study rDNA copy number heterogeneity among colonies, the presented data do not implicate such heterogeneities in the large discrepancies between CHEF- and WGS-based estimates.

Fourteen of the above strains were further assessed by whole genome sequencing, with two goals: 1) to verify the genotype of the given strains to eliminate the possibility that disparate copy numbers could be due to strain mislabeling, and 2) to assess how well WGS-based copy number estimates of yeast strains compare to CHEF-based results when both are performed in our lab. We generated yeast genomic DNA libraries of these 14 strains by Nextera tagmentation and amplification. WGS SNP analysis confirmed that genotype labels were correct for 11 of these 14 strains (Table S9, Figure 2C). BY4741 and B30 were included as known copy number controls. The resulting rDNA copy number estimates showed closer agreement with our CHEF-based results than the previously reported sequencing data, but were still highly divergent. Deviation from the CHEF-based copy numbers ranged from 2 to 38% (average 18%) when corrected for GC-content (Table 2). Overall, our yeast results reproduce the unreliability of rDNA copy number calls by Nextera WGS that we observed in *C. elegans*.

### Droplet digital PCR of yeast isolates

ddPCR has been proposed as a more precise alternative to qPCR (Hindson *et al.* 2013). ddPCR involves independent amplification of single molecules of target DNA, informing a Poisson distribution-based calculation of the absolute number of starting molecules (Vogelstein and Kinzler 1999; Hindson *et al.* 2013). ddPCR has been employed in rDNA copy number quantification with reported high success and has recently been used to estimate rDNA copy number in both human and yeast samples (Alanio *et al.* 2016; Xu *et al.* 2017; Salim *et al.* 2017). We wanted to assess ddPCR reproducibility and determine if ddPCR rDNA copy number estimates agree with CHEF-based estimates for a subset of *S. cerevisiae* wild isolate strains. We used ddPCR to quantify rDNA copy number in six strains (Table 2, Table S10), as well as the laboratory strain BY4741 (170 rDNA copies) and a low rDNA copy number strain, B30 (35 rDNA copies). BY4741 and B30 both have CHEF-based rDNA copy number estimates (Figure 2, Fig S1). Using published primer and probe sequences to target the rDNA (Salim *et al.* 2017), we optimized ddPCR conditions by testing and altering annealing temperature (Fig S2). We quantified both the number of rDNA copies and a single copy gene, *TUB1*, in the same ddPCR reaction and estimated copy number using Quantasoft software.

Estimates of rDNA copy number by ddPCR differed from CHEF-based estimates by 11–41% (average 22%)(Figure 2D, Table 2). For all samples, ddPCR underestimated copy number. The ddPCR calculation provides confidence intervals based on the Poisson distribution. For the three replicates of a given strain, the estimates did not always fall inside each others’ confidence intervals. Overall, ddPCR was nearly as technically reproducible as CHEF gel analysis; however, it yielded different, lower copy number estimates (CHEF- and ddPCR-based estimates were significantly different by *t*-test for all samples, ranging in p-value from 0.0006 to 0.011).

### Molecular inversion probe-based copy number estimation yields high error rates

**Figure 3:**
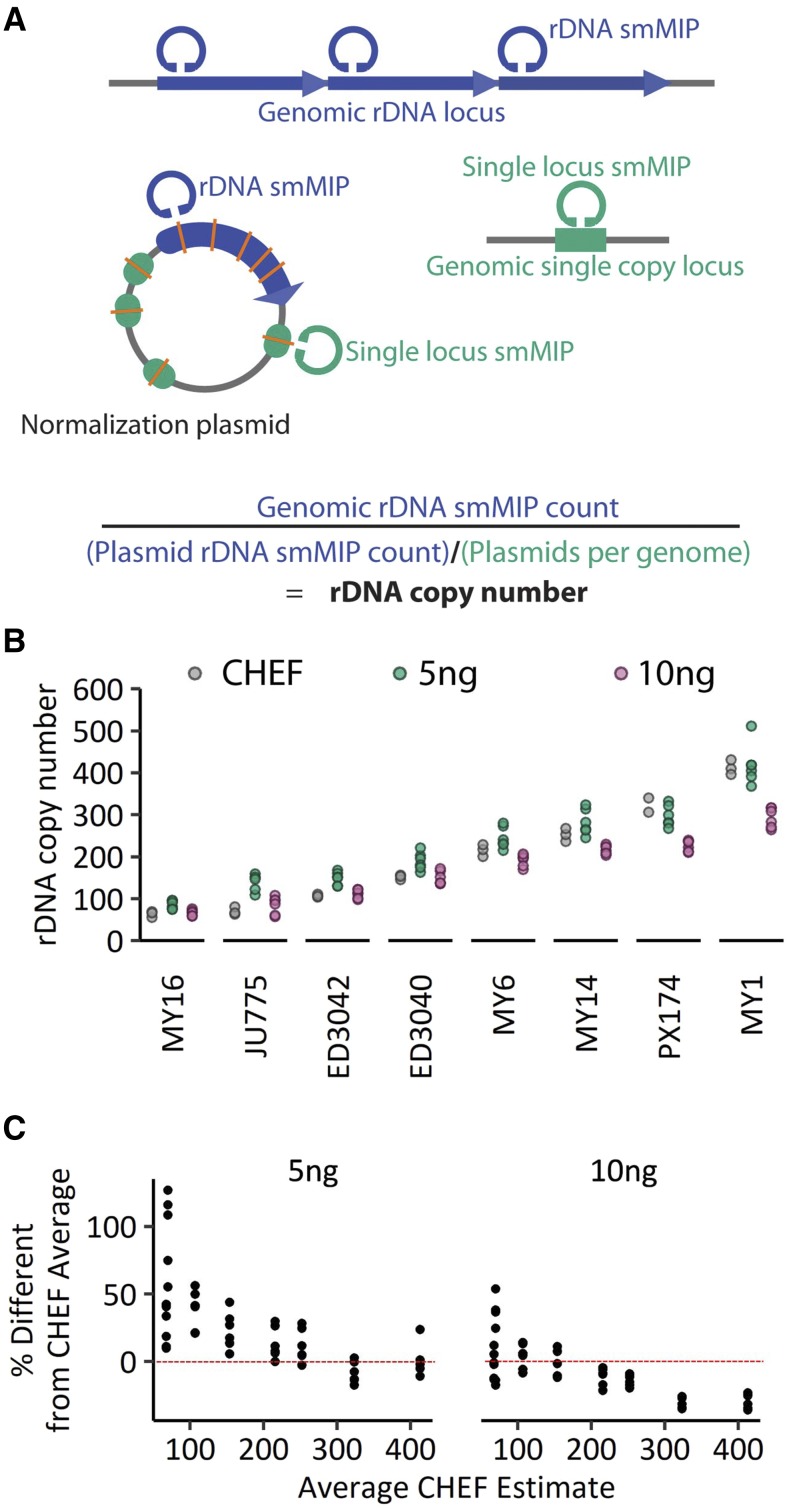
(A) Single molecule Molecular Inversion Probes (smMIPs) were targeted against regions of the rDNA (blue) and against four different single copy loci in the genome (green). A normalization plasmid contained a single copy of the rDNA repeat unit as well as the sequences of the four single copy loci, all with unique nucleotide variants (orange lines), only the two most consistent of which were used in the final analysis. smMIPs capture ∼100-150bp sections of the target DNA, with each capture event individually barcoded. Single-read sequencing was used to identify the barcode of each smMIP and source of its captured DNA (plasmid or genomic). The number of plasmids per genome was calculated from the ratio of genomic single copy loci smMIP capture events to normalization plasmid capture events. The rDNA copy number was estimated from genomic rDNA capture events relative to plasmid rDNA capture events, corrected for number of plasmids per genome. In this way, variable efficiency of probe binding is normalized. (B) Molecular inversion probe-based copy number estimation was performed using different input DNA amounts: 5ng and 10ng. rDNA copy number estimations are plotted here for eight wild *C. elegans* strains, alongside their CHEF-based copy numbers (dark blue). (C) 5ng and 10ng input amounts are compared against the CHEF-based rDNA copy number for the eight strains. Data are plotted as a percentage of the average CHEF-based values ((estimated number – CHEF number)/CHEF number).

Having observed the high error rate and inefficiency of WGS, we asked if a targeted sequencing approach could improve copy number estimation while at the same time requiring fewer overall sequence reads. Molecular inversion probes (MIPs) offer a means to target specific loci for sequencing at high depth (Hiatt *et al.* 2013; Mok *et al.* 2017). Previously, single molecule MIPs (smMIPs) have been used to capture and sequence short tandem repeats, another class of repetitive DNA elements (Carlson *et al.* 2015). smMIPs enable enumeration of individual capture events. In light of the enormous size of the rDNA locus, for which whole locus capture is impossible, we employed smMIP to estimate rDNA copy number by counting the number of capture events at the rDNA locus. Each targeted locus is captured many times in a pool of DNA molecules, and a unique 12-bp tag on each probe allows precise tracking of the number of molecules that have effected a capture. The confounding factor of variability in smMIP binding efficiency is corrected with a normalization plasmid (Figure 3A, Fig S3). The normalization plasmid contains one copy of the rDNA sequence with select nucleotide changes, and short single-copy regions from the genome, also with identifying nucleotide changes. smMIPs that target rDNA regions and smMIPS that target the single copy loci are pooled in the same reaction with a mixture of plasmid and genomic DNA. The target regions are captured, sequenced, and mapped back to plasmid or genomic origin using the plasmid-specific SNVs. In this way, we generated ratios of abundance of plasmid relative to genome copies (using the single copy loci), and abundance of genomic rDNA copies relative to plasmid, allowing us to estimate rDNA copies per genome (Figure 3A, Supplemental Data).

We optimized conditions to test different input amounts of genomic DNA (5ng and 10ng) and different ratios of genomic DNA to normalization plasmid input. We find that a 1:19 molar ratio of genomic DNA to plasmid gave us the best accuracy relative to CHEF-based estimates (Fig S3). Reminiscent of the WGS data (Figure 1C, D), estimates for high rDNA and low rDNA copy number strains were not necessarily affected in the same way; at 5ng input DNA, low copy number strains showed overestimated copy numbers while high copy number strains showed underestimated or close to predicted copy numbers (Figure 3B, C). Input DNA amount also affected copy number estimates (Figure 3B, C, Fig S3, Table S11). At 5ng input amount, JU775 averaged 97% above CHEF-based copy number, while MY1 averaged 1.5% above. At 10ng input amount, JU775 averaged 20% above CHEF-based copy number, while MY1 averaged 29% below. At 5ng input, the rDNA copy number estimates for the eight wild isolates differed anywhere from 0.3–127% (average 30%) from CHEF-based values. The targeted sequencing approach therefore failed to substantially improve upon the accuracy or reproducibility of whole genome sequencing. It is worth noting that we assessed smMIPs in the context of the small 100Mb genome of *C. elegans*. In the case of a much larger genome, such as the 3,000Mb genome of humans, targeted sequencing may still offer significant benefit over whole genome sequencing, if comparable accuracy can be obtained for many fewer reads.

### FISH-based rDNA copy number estimation shows promise for copy number estimates in individual cells

One future ambition for the field is to quantify rDNA copy number on a per-cell basis to better address potential tissue-specific and cell-specific variation in copy number. The methods we have thus far described were applied to pools of individuals. For larger organisms (*e.g.*, flies, mice, humans, plants), enough tissue can be obtained from a single individual to apply these methods, but cellular resolution is still desirable. Fluorescence *in situ* Hybridization (FISH) is a commonly used method to visualize genomic segments within intact cells. rDNA FISH has been done extensively, often to localize the rDNA or identify how many rDNA loci are present (Vitturi *et al.* 1999; Fontana *et al.* 2003; Garcia *et al.* 2017; Araújo da Silva *et al.* 2019). Others have used FISH to visualize 45S rDNA and thereby assess variability in rDNA content between cells (Rosato *et al.* 2016). However, these FISH methods did not allow for a quantitative estimation of rDNA copy number. To investigate if FISH signal intensities could serve as a readout for DNA copy number, we adopted a novel oligo-based FISH method (Supplemental methods, under review) that fluorescently labels the rDNA by tiling individual oligos along ten user-defined rDNA genomic sequences *in situ*. In contrast to prior FISH methods, this method inherently possesses single-molecule precision and is thus amenable for quantitative evaluation. We applied this method along with custom image analysis routines to detect rDNA FISH foci and quantify rDNA copy number in six of our thoroughly-characterized *C. elegans* wild isolates with CHEF-estimated rDNA copy numbers of 70, 107, 154, 216, 323, and 412. For ease of single cell retrieval, we used mixed embryonic stage worms instead of differentiated larvae or adults. Although cells were not synchronized, which may contribute to cell-to-cell variability within samples, we assume that the distribution of cell stages is not biased among the strains and thus strain characteristics can be compared on the population level.

**Figure 4:**
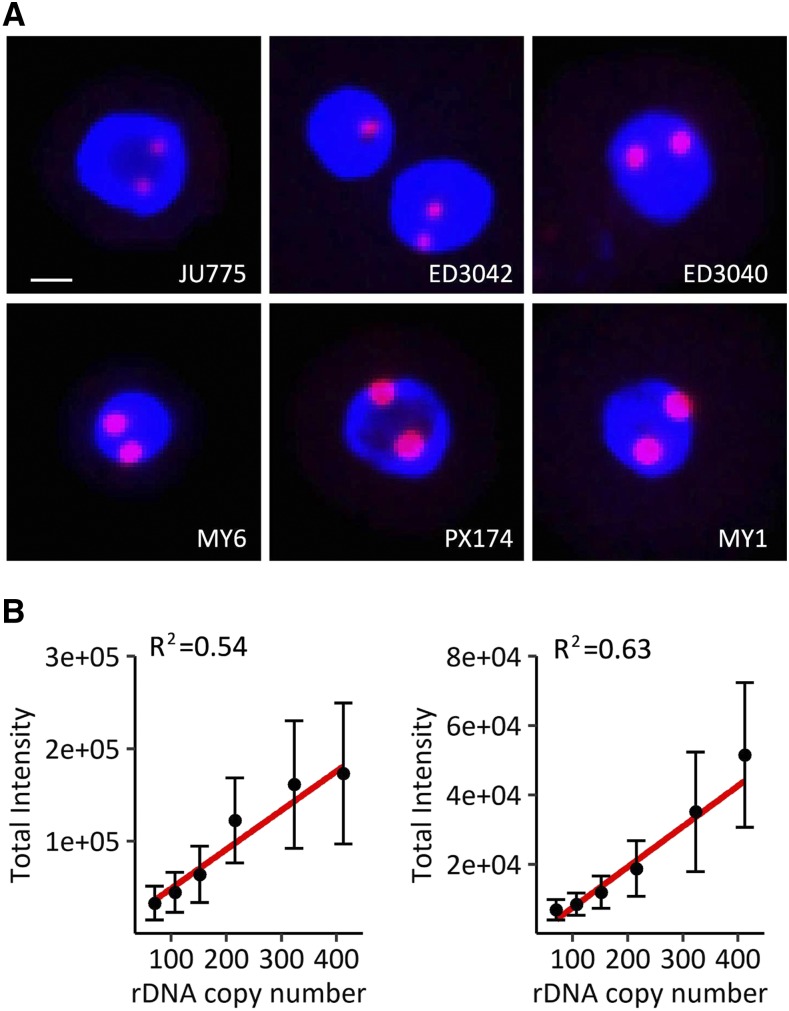
(A) Representative rDNA FISH images — rDNA FISH probe (red) and DAPI (blue) to stain nuclear DNA — in dissociated embryonic cells of six wild isolates *of C. elegans*. CHEF-based rDNA copy numbers of strains are: JU775 (rDNA 70), ED3042 (rDNA 107), ED3040 (rDNA 154), MY6 (rDNA 216), PX174 (rDNA 323), MY1 (rDNA 412). Scale bar equals 1μm. (B) Total intensity of each identified rDNA spot is plotted against the CHEF-based rDNA copy number for each strain, for replicate 1 (left, N ≥ 344) and replicate 2 (right, N ≥ 148 per strain).

In the majority of cells, we observed one or two distinct fluorescent foci per cell (Figure 4) (an average of 1.76 foci per cell over all samples), consistent with reported nucleolar marker fibrillarin localization during embryogenesis (Lee *et al.* 2012). For each strain, we imaged at least 100 cells (Table S12) and quantified the FISH foci in each cell. Upon analysis of the FISH foci intensities from all strains, we found that the integrated FISH spot intensity was concordant with their CHEF-based rDNA copy number (Figure 4, Fig S4, Table S13). The observed correlation between FISH spot intensity and rDNA copy number suggests that this method could be used to accurately estimate absolute copy number and its cell-to-cell variability in the presence of appropriate controls.

## Discussion

All scientific measurements have associated technical error. The strengths and weaknesses of different methods make them more or less suitable for a given task. The alarming discrepancy among multiple WGS-based copy number calls on a single genomic sample suggests that copy number data obtained from Nextera-enabled whole genome sequencing may not be accurate. Nevertheless, accurate knowledge of rDNA copy number variation has the potential to advance our understanding of complex traits including aging and cancer.

From our observations presented here, we propose several best practices to obtain the highest accuracy for rDNA copy number estimates. 1) If possible in a system, pulsed-field gel electrophoresis techniques should be employed to validate WGS-based copy numbers. 2) When applying whole genome sequencing, samples of validated copy number should be included in library preparations, to control for batch variation. 3) When applying whole genome sequencing or ddPCR, samples should only be compared that have been prepared and run together. 4) In the estimation of copy number from WGS data, GC-content computational correction should be performed (Benjamini and Speed 2012). To this end, our study presents a resource of yeast and worm strains with CHEF-gel verified rDNA copy number.

Historically, many methods not evaluated here have been used to quantify rDNA copy number, including 1) quantitative rRNA hybridization to total DNA (Schmickel 1973), to microarrays (Robyr *et al.* 2002; Carter 2007), or *in situ* to chromosomes (Henderson *et al.* 1972); 2) quantitative Southern blot (Kobayashi *et al.* 1998; Kwan *et al.* 2013); and 3) cytogenetic observations (Verma *et al.* 1977). Similarly, we did not discuss or explore more recent methods such as optical mapping (Lower *et al.* 2018). However, much of recent reporting of rDNA copy number variation has relied on estimation from short-read whole genome sequencing (Thompson *et al.* 2013; Gibbons *et al.* 2014, 2015; Xu *et al.* 2017; Wang and Lemos 2017; Parks *et al.* 2018). WGS-based copy number estimation is an attractive approach because of the vastness of existing data sets and the ease with which new data can be generated. Our observations, however, suggest that caution should be exercised when making conclusions from WGS data, especially when comparing libraries prepared at different times or by different facilities, even if using the same library preparation methods. Indeed, others have also observed library preparation batch drastically affecting copy number estimation (Li *et al.* 2018; Lofgren *et al.* 2019).

In the future, new technologies for estimating rDNA copy number may be available, including long-read sequencing such as developed by Oxford Nanopore, which can infrequently obtain read lengths of over 1Mb, with a record reported length of 2.27Mb (Payne *et al.* 2019). It should be noted that this read was reconstructed from eleven reads generated consecutively that mapped to a 2.27Mb locus (Payne *et al.* 2019), a gambit that may or may not be helpful for the case of long repetitive DNA, depending on the amount of sequence variation present among repeat units. Although researchers are working on extending the Nanopore readable length to make rDNA array size estimation practical, they face the likely obstacle of finding DNA extraction techniques gentle enough to recover fragments large enough to span the rDNA (Tyson *et al.* 2018). Recent long-read sequencing of *C. elegans* using both PacBio and Nanopore failed to determine copy number of either the 45S or 5S rDNA repeats (Yoshimura *et al.* 2019). For a *C. elegans* strain of 400 rDNA copies, covering the entire array would require reads of nearly 3Mb. Application in humans, with a single unit size of 43kb, will likely need read lengths upwards of 6Mb (Stults *et al.* 2008).

From the biomedical perspective, human rDNA copy number is of substantial interest, and error in rDNA copy number calls impairs our ability to incorporate this potentially valuable source of variation into association studies. It is likely that the problem of human rDNA copy number determination is even more challenging than our results here suggest, as the model organisms presented have only a single rDNA locus and are thus the simplest cases. Human 45S rDNA loci are spread across five chromosomes, making validation by CHEF gel less feasible due to the difficulty of interpreting multiple gel bands, some of which may be too large to easily resolve on a pulsed-field gel (Stults *et al.* 2008). FISH and other single-cell approaches may prove an alternative way to quantify copy number in humans, with the benefit of potentially providing cell and tissue level information.

We have presented here a method of FISH-based quantification as a future avenue for repetitive locus quantification. The application of tiling oligo-based FISH showed promise in worm cells of different strains, and future application to intact worms could elucidate rDNA variability among individuals, between tissues in an individual, and across life stages. In species with multiple rDNA loci, FISH approaches also have the potential to assign locus-specific copy numbers, a level of characterization so far unaddressed. Previous attempts used pseudo-quantitative methods like argyrophilic nucleolar organizing region staining (Héliot *et al.* 2000).

One caveat of our study is the necessity of designating a ‘true’ value for rDNA copy number. Herein, we have used CHEF-based estimates as our measure for comparisons. We do, however, acknowledge the possibility that CHEF-based measurements may be subject to electrophoretic artifacts. However, previous reports find either no or only minimal effect of GC content or repetitiveness on mobility in an agarose gel (Schaffer and Sederoff 1981, Elder and Southern 1983, Pourcel *et al*. 2011, De Bustos *et al*. 2016), giving us confidence in the accuracy of CHEF-based copy number estimates.

The observed difficulties in copy number estimation of the rDNA locus likely extend to other repetitive genomic loci, making this problem of relevance not just to rDNA but to the genome as a whole. It is our hope that the recommendations we outline will advance our ability to characterize this type of variation and promote its eventual incorporation into our understanding of biology.
